# Augmented Reality Integration in Skull Base Neurosurgery: A Systematic Review

**DOI:** 10.3390/medicina60020335

**Published:** 2024-02-16

**Authors:** Emir Begagić, Hakija Bečulić, Ragib Pugonja, Zlatan Memić, Simon Balogun, Amina Džidić-Krivić, Elma Milanović, Naida Salković, Adem Nuhović, Rasim Skomorac, Haso Sefo, Mirza Pojskić

**Affiliations:** 1 Department of General Medicine, School of Medicine, University of Zenica, Travnička 1, 72000 Zenica, Bosnia and Herzegovina; zlatan_memic@outlook.com; 2 Department of Neurosurgery, Cantonal Hospital Zenica, Crkvice 67, 72000 Zenica, Bosnia and Herzegovina; hakija.beculic@dl.unze.ba (H.B.);; 3 Department of Anatomy, School of Medicine, University of Zenica, Travnička 1, 72000 Zenica, Bosnia and Herzegovina; rpugonja@gmail.com; 4 Division of Neurosurgery, Department of Surgery, Obafemi Awolowo University Teaching Hospitals Complex, Ilesa Road PMB 5538, Ile-Ife 220282, Nigeria; 5 Department of Neurology, Cantonal Hospital Zenica, Crkvice 67, 72000 Zenica, Bosnia and Herzegovina; 6 Neurology Clinic, Clinical Center University of Sarajevo, Bolnička 25, 71000 Sarajevo, Bosnia and Herzegovina; 7 Department of General Medicine, School of Medicine, University of Tuzla, Univerzitetska 1, 75000 Tuzla, Bosnia and Herzegovina; naidasalkovic@gmail.com; 8 Department of General Medicine, School of Medicine, University of Sarajevo, Univerzitetska 1, 71000 Sarajevo, Bosnia and Herzegovina; ademnuhovic@gmail.com; 9 Department of Surgery, School of Medicine, University of Zenica, Travnička 1, 72000 Zenica, Bosnia and Herzegovina; 10 Neurosurgery Clinic, Clinical Center University of Sarajevo, Bolnička 25, 71000 Sarajevo, Bosnia and Herzegovina; 11 Department of Neurosurgery, University Hospital Marburg, Baldingerstr., 35033 Marburg, Germany

**Keywords:** augmented reality, skull base neurosurgery, neurosurgical training, neuronavigation, virtual reality

## Abstract

*Background and Objectives:* To investigate the role of augmented reality (AR) in skull base (SB) neurosurgery. *Materials and Methods:* Utilizing PRISMA methodology, PubMed and Scopus databases were explored to extract data related to AR integration in SB surgery. *Results:* The majority of 19 included studies (42.1%) were conducted in the United States, with a focus on the last five years (77.8%). Categorization included phantom skull models (31.2%, *n* = 6), human cadavers (15.8%, *n* = 3), or human patients (52.6%, *n* = 10). Microscopic surgery was the predominant modality in 10 studies (52.6%). Of the 19 studies, surgical modality was specified in 18, with microscopic surgery being predominant (52.6%). Most studies used only CT as the data source (*n* = 9; 47.4%), and optical tracking was the prevalent tracking modality (*n* = 9; 47.3%). The Target Registration Error (TRE) spanned from 0.55 to 10.62 mm. *Conclusion:* Despite variations in Target Registration Error (TRE) values, the studies highlighted successful outcomes and minimal complications. Challenges, such as device practicality and data security, were acknowledged, but the application of low-cost AR devices suggests broader feasibility.

## 1. Introduction

Neurosurgical procedures of various lesions on the base of the skull, such as the tumor removal or aneurysms repairing, are among the most often performed in clinical practice [1]. Afterwards, the reconstruction of the base of the skull is required, which decreases the development of possible complications, like infection or leaking of cerebrospinal fluid [2]. Hence, the techniques used by neurosurgeons have tremendously improved in recent years, especially with the development of endoscopic surgery and innovations in neuroimaging. During every neurosurgery procedure, the main focus for the surgeon is to accurately and precisely target specific anatomical structures [3,4]. This can be a potential obstacle in the operation field due to complex anatomic relationships of all structures in that small, tight area of the cranial base, which can cause various complications, such as the lesions of surrounding vascular or nerve structures [5]. Therefore, novel advanced techniques that enable the surgeon to assess the anatomy details of every patient by the use of neuronavigation (NN) are currently being investigated. However, there are still many challenges in clinical practice. For example, neurosurgeons currently use a virtual environment based on the pre-operative 3D images and radiological data of patients that are then displayed on the 2D screen [6]. Afterwards, surgeons are required to shift their focus from that screen back to the surgical field, comparing and matching the real operating field to the virtual one and leaving a lot of room for mistakes. In addition, there is a decrease in the precision and increase in the duration period of the neurosurgery [7]. Augmented reality (AR) is among the novel techniques that aim to solve these important obstacles. AR was introduced in the science field many years ago, but its engineering and application in neurosurgery and in other surgical branches (e.g., maxillofacial surgery, plastic surgery, general surgery, etc.) started promptly and progressively since 1995 [8]. It is a technique that combines images generated by software with real visual and surgical data, leading to an enhancement in the neurosurgeon’s visual experience. During the surgery, AR enables merging the images taken before surgery and real-time intraoperative imaging data [8,9,10].

AR headsets and smart glasses, utilized by surgeons, transform the visual field by superimposing virtual and real data [11]. Engineered to project 3D images of a patient’s anatomy, they aid in procedure planning and neurosurgical training [12]. While current AR setups are costly, advancements in smartphones and tablets with 3D display capabilities offer a potential cost-effective alternative [13,14]. In skull base surgery, a proposed AR model involves displaying endoscopic images centrally and projecting virtual images externally using previously collected patient data, enabling enhanced integration and refinements in neuroimaging and NN [14,15,16].

This systematic review aims to summarize current knowledge on the use of AR in SB surgery. Although AR has the potential to develop into a ground-breaking technique that would transform neurosurgical procedures into minimally invasive and safe, many unknowns are still not deciphered. The technical success and shortcomings of the so-far used systems and their features (e.g., the virtual data source, tracking modality, registration technique and display type) in the in vivo settings on humans are discussed in this systematic review. In addition, a comparison of the practical application of this ground-breaking method in various countries has been researched.

## 2. Materials and Methods

### 2.1. Study Methodology and Registration

A thorough and systematic examination was undertaken to evaluate the current utilization of AR in SB surgery. The methodology adhered to the established guidelines of PRISMA (Preferred Reporting Items for Systematic Reviews and Meta-Analyses). Registration of this systematic review took place in the Open Science Framework (OSF) registry, and it is identified by the unique identifier OSF-REGISTRATIONS-HPZ4S-V1.

### 2.2. Search Strategy

On 12 September 2023, a literature search for English-text articles was performed using PubMed and Scopus. In PubMed, the key terms “augmented reality” and “skull-base” were employed for the search. The same search strategy was applied to the Scopus database. For a more detailed presentation of the search strategies, refer to Appendix A, which contains pertinent information related to the data retrieval process. The confirmation of adherence to the PRISMA methodology was carried out using the PRISMA checklist, as presented in Appendix B.

### 2.3. Inclusion and Exclusion Criteria

**Figure 1:**
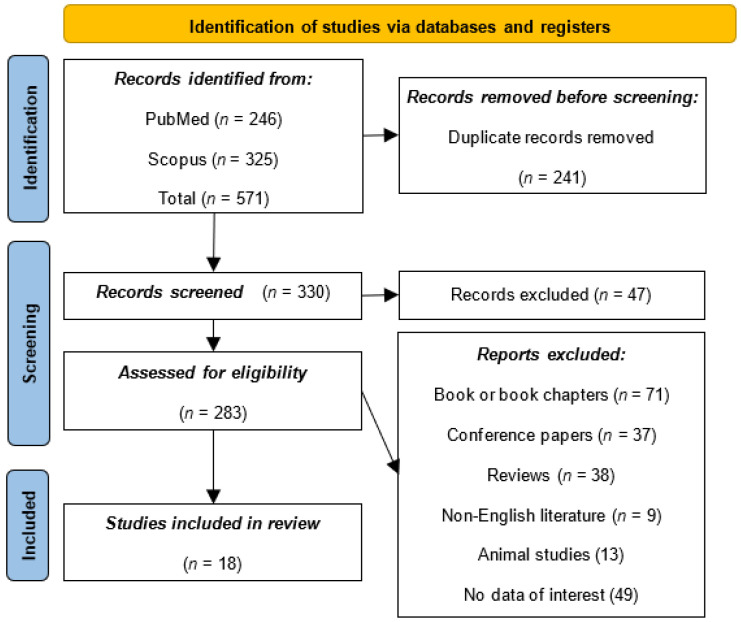
PRISMA flowchart.

The inclusion criteria for this study involved selecting articles that were written in English, directly pertinent to the intersection of AR and SB surgery, and possessed relevant data addressing the study’s objectives. This ensured a focused and language-consistent examination of the subject matter. The exclusion criteria aimed to refine the selection by excluding book chapters, conference papers, reviews, non-English literature, animal studies, and articles without data of interest. The PRISMA flowchart provides a systematic overview of the article selection process for this review (Figure 1). Initially, a total of 571 records were identified from two databases: PubMed (*n* = 246) and Scopus (*n* = 325). Before the screening process, 285 duplicate records were removed, resulting in 285 unique records subjected to screening. During the screening phase, 47 records were excluded based on predefined criteria. The remaining 215 reports were assessed for eligibility, leading to the exclusion of additional articles based on specific criteria, such as book or book chapters (*n* = 71), conference papers (*n* = 37), reviews (*n* = 38), non-English literature (*n* = 9), animal studies (*n* = 13), and articles lacking data of interest (*n* = 49). Ultimately, 19 studies met the inclusion criteria and were included in the review.

### 2.4. Data Extraction

In conducting this systematic review, a meticulous approach to data extraction was undertaken to capture essential details pertaining to each included study. The extracted information encompassed various critical elements that collectively contributed to a nuanced understanding of the intersection between AR and SB surgery.

The quantitative aspects of the review were addressed through the extraction of sample size (N), enabling an assessment of the scale and scope of the studies. Furthermore, the identification of specific anatomical targets, treated pathologies, surgical modalities, virtual data sources, tracking modalities, registration techniques, display types, and Target Registration Error (TRE) offered a comprehensive snapshot of the diverse applications and technologies employed in the integration of AR within SB surgical procedures.

### 2.5. Statistical Analysis

Basic statistics were employed to present frequencies and absolute numbers, providing a quantitative overview of various critical elements related to the utilization of AR in SB surgery. To enhance the presentation and interpretation of the findings, graphical visualization was performed using Microsoft Excel (v. 2021, Microsoft Corporation, Washington, DC, USA).

## 3. Results

### 3.1. Characteristics of Included Studies

**Figure 2:**
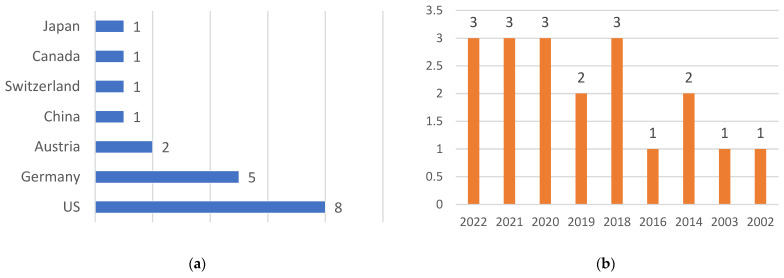
Geographical and spatial distribution of included studies: (**a**) geographical distribution; (**b**) temporal distribution.

The data derived from the analysis of 19 selected studies that fulfilled the inclusion criteria revealed that a predominant proportion of these studies originated from the United States, constituting 42.1% of the total (*n* = 8), with Germany contributing the second highest at 26.3% (*n* = 5) (Figure 2a). The temporal distribution indicated that the majority of the studies, amounting to 77.8% (*n* = 15), were conducted within the past 5 years (Figure 2b). The subjects under investigation in the recruited studies were categorized as either 3D-printed phantom skull models (31.2%, *n* = 6), human cadavers (15.8%, *n* = 3), or human patients (52.6%, *n* = 10). Notably, Li et al. [16] employed both phantom skull models and cadavers in their research.

Microscopic surgery was the predominant modality among the included studies, used in 10 studies (55.8%). Of the 19 included studies, surgical modality was specified in 18. Microscopic surgery was the predominant modality among the included studies, used in 10 studies (52.6%). Among the included studies, the majority used only CT as the data source (*n* = 9; 47.4%), two (10.5%) studies used only MRI, and five (31.6%) used both CT and MRI. Two (10.5%) studies did not specify the data source. Optical tracking was the predominant tracking modality used by the studies (*n* = 9; 47.3%).

### 3.2. Study Sample Characteristics

Among the studies under consideration, Bopp et al. [17] conducted the largest investigation, featuring a sample size of 164 participants. Subsequently, Zeiger et al. [18], Carl et al. [19], Schwam et al. [20], and Pojskić et al. [21] followed with sample sizes of 134, 47, 40, and 39, respectively. While the sample sizes for two studies were unspecified, the cumulative sample size from the 19 studies that provided this information amounted to 508.

### 3.3. Results from Laboratory Studies

In the laboratory studies on phantom skull models and cadaver studies, the accuracy of AR in defining normal anatomy of the SB structures or in the surgical approach to the SB was assessed. However, in a study by Steiert et al. [22], defects were artificially created on the fronto-orbital area of the phantom skull models with extension to the SB, and a reconstruction of this defect was undertaken with AR. TRE values within the presented studies reveal notable variations in the accuracy of surgical navigation across different methodologies and virtual procedures. Lai et al. [23] in the USA achieved a relatively low TRE of 0.55 ± 0.24 mm for procedures involving the sphenoid sinus and pituitary gland, employing an endoscope and Cone-Beam Computed Tomography (CBCT). In contrast, Li et al. [16] in China reported a slightly higher TRE of 1.28 ± 0.45 mm for similar procedures using a combination of CT, Optical Tracking System (OTS), and manual registration. Birkfellner et al. [24] in Austria, focusing on a skull model, demonstrated a TRE of 0.9 mm with a micro tracking system and Optical Tracking System (OTS) using fiducial markers (Table 1).

### 3.4. Results from Clinical Studies

AR studies on humans were undertaken to operate surgical pathologies of the SB in 292 patients. Further, eight of the nine studies were specific on the pathology operated with AR for 252 patients. A study by Schwam et al. [8], which involved the use of AR for the operation of 40 SB tumors, did not specify the pathology operated. Of 416 specified, pituitary adenoma was the predominant pathology, which accounted for 68.3% (*n* = 284). This was followed by SB meningioma (13.2%, *n* = 55) and Rathke cleft cyst (1.4%, *n* = 6). TRE values within the specified studies present a spectrum of precision in surgical navigation for various pathologies and treatment modalities. Notably, Pojskić et al. [21] in Germany achieved a relatively low TRE of 0.82 ± 0.37 mm for 39 skull-based meningiomas using a micro tracking system with a hybrid anatomical landmark and surface matching registration technique. Bopp et al. [17] noted TRE values of 0.76 ± 0.33. In contrast, Carl et al. [19] in Germany reported a higher TRE of 2.33 ± 1.30 mm for adenoma surgeries, employing microsurgical techniques with fiducial markers and an electromagnetic navigation (EMN) system (Table 2).

### 3.5. Results from Cadaveric Studies

The presented studies offer insights into the Target Registration Error (TRE) within different surgical navigation approaches. Leuze et al. [29] in the USA, focusing on an 8-case retrosigmoid approach, utilized a microsurgical modality with Cone-Beam Computed Tomography (CBCT) as the virtual data source. They employed an optical tracker and fiducial markers, with manual registration. McJunkin et al. [30] in the USA, with an unspecified number of cases and a generic target, employed CT and MRI as the virtual data source. They utilized an Optitrack tracking modality with surface matching registration, achieving a TRE of 5.76 ± 0.54 mm. Dixon et al. [31] in Canada conducted a transsphenoidal SB approach, utilizing an endoscopic modality with CT as the virtual data source. They implemented an Optical Tracking System (OTS) with fiducial markers, achieving a TRE of 2.6 mm, and utilized an AR display (Table 3).

## 4. Discussion

In this systematic review, 18 studies that focused on the goal of this research were included. We aimed to summarize current knowledge on the use of AR in SB surgery. The results showed that the majority of the studies that met the inclusion criteria were from the US, accounting for 47% of the studies, followed by Germany, which accounted for 20%. The results align with the findings of the systematic review conducted by Ismail Zaed et al. [32]. Ismail Zaed et al. [32] stated that 27.3% of studies examining the applicability of AR in neurosurgery, in general, were conducted in the US. In the previously mentioned studies, the second and third places were taken by Germany and China, which differs from our results, in which the third country with the most extensive number of studies is Austria. The US still holds the title as the most innovative in the medical technology industry, and the reasons behind this trend are the highest amount of investment in innovative neurosurgical technology and the highest research activity in neurosurgery. According to Sarica and Egemen [33], the US emerged as the leading contributor, accounting for 35% of the publications in the field of neurosurgery.

Deng et al. [34] outlined the integral role of Image-Guided Neurosurgery Systems (IGNs) in neurosurgery. These systems utilize preoperative patient images, allowing surgeons to monitor the tumor’s relative position in real-time during procedures and to clearly delineate the tumor’s edges. However, they highlighted a significant challenge: surgeons must frequently switch their focus between the computer screen and the surgical field to reconcile their relative position with the preoperative brain images. This necessity underscores the evolving role of AR in enhancing surgical precision and efficiency. Expanding upon this notion, Sik-Lanyi et al. [35] articulated that neurosurgery, as a medical discipline, is profoundly reliant on imaging methodologies. They posited that Augmented Reality (AR) harbors the transformative potential to revolutionize and reshape the methodologies through which neurosurgeons strategize and execute surgical procedures in the future. Kersten-Oertel et al. [36] designed an advanced AR system specifically for arteriovenous malformation (AVM) surgery. This system integrates seamlessly with the surgical microscope, employing chromadepth rendering and blood vessel color coding. The chromadepth feature provides vital depth information about blood vessels in relation to the brain’s surface. More importantly, the color coding aids in distinguishing arterial and venous blood vessels, a crucial factor in AVM surgery due to the similarity in the appearance of arterialized blood vessels [37]. Additionally, the AR system’s alpha blending control allows surgeons to adjust the translucency of the overlaid virtual image, demonstrating the potential of AR in enhancing the visualization capabilities in highly specialized neurosurgical procedures [9].

Further illustrating the versatility of AR in neurosurgery, Abe et al. [38] conducted experiments using the Virtual Protractor with AR (VIPAR) system. This research, initially carried out on 40 spine phantoms in a laboratory setting, showed significant improvements in the accuracy of vertebroplasty needle insertion angles compared to traditional methods. Subsequently, the VIPAR system was applied in a clinical setting at Enewa Hospital (Enewa, Hokkaido, Japan), where it assisted in five successful percutaneous vertebroplasty procedures without any complications, like spinal pedicle breach or cement leakage. These findings highlight AR’s effectiveness in guiding neurosurgeons during complex procedures, offering a glimpse into the future of AR applications in subspecialties like spinal surgery.

Presently, low-cost AR devices show significant promise for surgery [39,40,41,42]; however, there is a lack of reports on their technical feasibility [43,44,45]. Kubben and Sinlae [43] conducted a pilot study to assess the feasibility of using the Microsoft HoloLens, a low-cost head-mounted holographic AR device, in SB neurosurgery. The device, controlled through hand gestures, enables “touch-free” operation in a sterile setting. The results showed comfort in wearing, accurate gesture recognition under various conditions, and effective voice recognition. The authors concluded that this commercially available AR device is practical for neurosurgery, offering versatility in different surgical environments and anticipating new opportunities for image-guided surgery.

AR demonstrated broad applications in neurosurgery, particularly in minimally invasive procedures, due to its enhancement in orientation in the surgical field [32,46,47,48]. This technology leads to a reduction in complications and enables more extensive tumor resection [49,50]. The included studies present a wide spectrum of pathological conditions at the SB that have been successfully treated with the assistance of AR, including craniopharyngioma, abscess, fibrous tumor, aneurysmatic bone cyst, germinoma, Rathke cleft cyst, osteochondromyxoma, iatrogenic CSF leak, myxoma, GH-secreting adenoma, papillary craniopharyngioma, meningiomas, pituitary adenomas, chordoma, and CSF leaks. Overall, the primary areas of neurosurgery where AR is most extensively applied include spinal surgery, NN, and education [6,51]. Its application is also beneficial in hydrocephalus surgery [52], neuroimaging [53,54], surgery for brain tumors [55], cerebrovascular pathology [56], ventricular system surgery [57], neurotrauma [58], and neurodegenerative pathology [59].

According to Pennacchietti [26], radical tumor resection was achieved in 65% of cases without fatalities and long-term complications. Pojskić et al. [21] reported that there were no injuries to critical neurovascular structures during surgery, with a TRE of 0.82 ± 0.37 mm.

In a study by Lai et al. [23], the accuracy of overlap, measured using TRE, was 0.55 ± 0.24 mm, while in a cadaveric study by Li et al. [16], a TRE of 1.28 ± 0.45 mm was demonstrated. McJunkin et al. [30] reported an average TRE of 5.76 ± 0.54 mm, indicating notable variability in the implementation of AR within neurosurgery. In a study by Creighton et al. [25], the observed TRE was 10.62 ± 5.90 mm. Dixon et al.’s [60] investigation revealed no significant difference in the median TRE between the groups employing AR and the control groups (2.9 vs. 2.6 mm). Citardi et al. [61] put forth the proposition that the objective for the next-generation surgical navigation platform should be to diminish TRE to 1.0–1.5 mm or, ideally, to 0.6–1.0 mm. The TRE significantly depends on various factors, including access to anatomical registrations, imaging modalities, and tracking approaches [62,63,64]. Consequently, comparing TRE values between studies with different designs lacks practical significance. Therefore, the TRE values should be compared to the target values [61].

Zeiger et al. [18] reported no intraoperative complications in a series of 134 cases using an AR setup. In the study by Pojskić et al. [21], perioperative surgical complications occurred in six patients (15.4%), including cerebrospinal fluid leakage requiring surgical intervention, wound healing disorders necessitating revision, and postoperative hydrocephalus with ventriculoperitoneal and subdural-peritoneal shunt implantations. Regarding the transsphenoidal approach in conventional neurosurgery, Laws et al. documented 24 vascular complications in a series of 3061 transsphenoidal operations for pituitary pathology, with 9 being fatal. Ciric et al. [65] reported an overall mortality rate of 0.9% for such operations and an incidence of severe complications, such as carotid artery damage or vision loss, ranging from 1 to 2%. Through a meticulous analysis of these findings, it is apparent that the incidence of postoperative and perioperative complications is comparable between AR approaches and conventional modalities.

Radical resection requires thorough preoperative preparation by neurosurgeons, especially when the lesion is located in challenging intracranial areas [66,67,68,69]. The application of AR technology in this context has significant implications for SB surgery, enabling precise surgical planning and achieving radical resection in certain neoplasm forms such as gliomas [68,69,70,71]. Given the complexity of SB surgery in neurosurgery, educational challenges for young neurosurgeons are common [72]. The use of AR has facilitated education in intracranial pathological entities in surgery overall [73,74,75]. Gestel et al. [76] confirmed its educational potential in the planning process of neurosurgical procedures. Additionally, Sommer et al. [77] noted that the integration of AR can facilitate workflow, especially in cases involving complex anatomy. However, they emphasize the need for simplifying the interaction with software that serves as an intermediary between AR and surgeons. In other words, the software should be more user-friendly.

The use of AR in SB surgery is at a specific stage of development. On the one hand, there are obvious advantages that real-time 3D technology offers to surgeons in optimizing procedures related to SB surgery [14,78,79,80]. On the other hand, this promising technology currently has its limitations, which are reflected in the current development of the equipment itself [81], the training of staff to use these new techniques [82], and the inaccessibility in less-developed countries or countries whose health systems are less able to follow the technological development of medical services [83]. Thavarajasingam et al. [84] concluded, in a systematic analysis of 17 papers related to the use of AR in transsphenoidal surgery, that AR provides a convincing improvement to landmark identification, intraoperative navigation, and surgeon experience, with a positive effect on accuracy and time. They also emphasize the limited number of published studies regarding the topic, which limits further conclusions. Li-Ming et al. [85] noted the potential benefits of using augmented or mixed reality devices to improve the precision of tumor margin excision. The same research, however, emphasized the negative sides of using new technology in that period of development; the technologies developed were too bulky, or they interfered with sterilization, lacked depth perception, or had other significant limitations, which restricted their use in operations. Kalavakonda et al. [86] built upon this research, with AR application for aiding tumor resection in SB surgery. They developed software to input DICOM CT images and output live 3D overlays to the HoloLens device, using Unity 3D Game Engine as the recommended platform for developing applications on the HoloLens. Also, they used an iso-surface visualization algorithm to convert two-dimensional images into a 3D volume by generating a mesh with triangles of constant density. Although this demonstrates an obvious improvement in technological capabilities compared to earlier research, the authors, nevertheless, emphasized limitations that still need to be addressed in the future. This primarily refers to reducing mesh size, which can still be too large for mobile devices to handle, and the development of more advanced algorithms that would enable faster and more natural interaction in real time [87]. The current literature recognizes several technical limitations in the use of AR, primarily the impracticality of the devices surgeons uses. Limited battery life [88], large devices, and impractical cables limit the potential of this technology at the present time [89]. Another challenge of using devices which are essentially turning patients’ information into virtual reality is safely storing that information in electronic records, which requires the integration of recordable HMDs into medical practice and securing data according to national and international data protection laws [81].

Future directions in spinal and neurosurgery should prioritize addressing technical limitations in Augmented Reality (AR) devices, including battery life and practicality. Efforts must focus on improving the user-friendliness of AR software and developing advanced real-time interaction algorithms. The standardization of AR applications across surgical environments is crucial for practical feasibility. Collaboration between the medical technology industry and neurosurgical community is key for innovation, aiming to reduce the Target Registration Error (TRE) to the proposed benchmarks. Additionally, efforts should be dedicated to advancing AR-based educational initiatives for young neurosurgeons, addressing challenges in training for complex intracranial surgeries.

This study’s limitations include the potential exclusion of relevant literature post 12 September 2023, exclusion of non-English literature, book chapters, conference papers, and animal studies. Additionally, a notable limitation is the small number of included studies (19), which may impact the generalizability and robustness of the findings.

## 5. Conclusions

In conclusion, this systematic review provides insights into the current status of AR applications in SB surgery. The majority of research originates from the US, aligning with global trends in neurosurgical innovation. The findings highlight AR’s transformative potential in enhancing precision, particularly in spinal surgery, neuro-navigation, and education. Noteworthy studies emphasize successful outcomes and minimal complications, despite variations in TRE values. Challenges, such as device practicality and data security, are acknowledged, but the application of low-cost AR devices suggests broader feasibility. Despite technical hurdles, AR emerges as a promising and evolving tool in neurosurgery, poised for further innovation and refinement.

## Figures and Tables

**Table 1:** Data summary from laboratory studies [15,16,22,23,24,25].

Reference	Year	Country	N	Target	Treated Pathology	Surgical Modality	Virtual Data Source	Tracking Modality	Registration Technique	Display Type	TRE or FRE (mm)
Lai et al. [23]	2020	USA	1	SP	Sphenoid Sinus and Pituitary gland	Endo	CBCT	OTS	OTS	Endoscope display	0.55 ± 0.24
Li et al. [16]	2016	China	9 +15	9 SP + 15 C	Sphenoid Sinus and Pituitary gland	Endo	CT	OTS	Manual	LCD (workstation)	1.28 ± 0.45
Steiert et al. [22]	2022	Germany	9	SP	Skull defect implantations (Fronto-orbital with extension to anterior skull base) (PMMA)	Micro	CT	N/d	Manual	HDM	N/d
Bong et al. [15]	2018	USA	7	SP	Trans-phenoidal Anatomy	Endo	N/d	OTS	OTS	Monitor	~1
Creighton et al. [25]	2020	USA	1	SP	Skull Model	Endo	CT	Hololens	Hololens	HMD	10.62 ± 5.90
Birkfellner et al. [24]	2003	Austria	1	SP	Skull Model	Micro	CT	OTS	Fiducial markers	HDM	0.9

Legend: SP—skull phantom; OTS—Optical Tracking System; CBCT—Cone-Beam Computed Tomography; CT—Computed Tomography; Endo—Endoscopic; N/d—Not specified; HDM—Head-Mounted Display; LCD—Liquid Crystal Display; TRE—Target Registration Error; FRE—Fiducial Registration Error.

**Table 2:** Data summary from clinical studies [13,17,18,19,20,21,26,27,28].

Reference	Year	Country	N	Target	Treated Pathology with AR	Surgical Modality	Virtual Data Source	Tracking Modality	Registration Technique	Display Type	TRE
Pennacchietti et al. [26]	2021	Germany	17	P	Craniopharyngioma, abscess, fibrous tumor, aneurysmatic bone cyst, germinoma, Rathke cleft cyst, osteochondromyxoma, Iatrogenous CSF leak, myxoma, GH-secreting adenoma, and papillary Craniopharyngioma	Endo	MRI	Optical reference frame for the endoscope	Hybrid anatomical landmark and surface mesh	Endoscope display	N/d
Pojskić et al. [21]	2022	Germany	39	P	39 skull-based meningiomas	Micro	CT+MRI	Hybrid anatomical landmark	Surface matching registration	HUD	0.82 ± 0.37
Schwam et al. [20]	2021	USA	40 (nearby, n/d)	P	SB tumors (N/d)	Micro	N/d	BrainLab Curve™ and Surgical Theater	N/d	HUD	N/d
Barber et al. [27]	2018	USA	1	P	Cystic mass in the left petrous apex	Endo	CT	Viva trackers	3D printed model	N/a	N/d
Barber et al. [27]	2018	USA	1	P	Transnasal cyst mass drainage	Endo	CT	Viva trackers	3D printed model	Otoendoscope	N/d
Kawamata et al. [28]	2002	Japan	12	P	12 pituitary adenomas	Endo	CT+MRI	Optical system	Optical	Endoscope monitor	N/d
Zeiger et al. [18]	2020	USA	134	P	68 pituitary tumors (68), meningiomas (16), Rathke’s cleft cyst (10), CSF leaks (3)	Endo	CT+MRI	OTS	Fiducial markers, automatic iCT	Endoscope monitor	N/d
Bopp et al. [17]	2022	Germany	164	P	Adenoma (81)	Endo	CT+MRI	Anatomical landmarks	Fiducial markers (iCT)	HUD	0.76 ± 0.33
Carl et al. [19]	2019	Germany	47	P	Adenoma (43), biopsy (4)	Micro	MRI, CT, RF	EMN	Fiducial markers	HUD	2.33 ± 1.30
Cabrilo et al. [13]	2014	Switzerland	1	P	Inferior Clivus Cordoma	Micro	N/d		Surface matching system	N/d	N/d

Legend: N—study sample, CSF—Cerebrospinal fluid; N/a—not available; MRI—Magnetic Resonance Imaging; N/d—Not defined; HUD—Heads-Up Display; OTS—Optical Tracking System; CT—Computed Tomography; iCT—intraoperative Computed Tomography; TRE—Target Registration Error.

**Table 3:** Data summary from cadaveric studies [29,30,31].

Reference	Year	Country	N	Target	Treated Pathology	Surgical Modality	Virtual Data Source	Tracking Modality	Registration Technique	Display Type	TRE
Leuze et al. [29]	2021	USA	8	C	Retrosigmoid approach	Micro	CBCT	Optical tracker	Fiducial markers, manual	HMD	N/d
McJunkin et al. [30]	2019	USA	N/d	C	N/a	N/a	CT+MRI	Optitrack	Surface matching registration	HoloLens MR headset	5.76 ± 0.54
Dixon et al. [31]	2014	Canada	1	C	Transsphenoidal SB aproach	Endo	CT	OTS	Fiducial markers	AR display	2.6

Legend: N—study sample; CBCT—Cone-Beam Computed Tomography; N/a—not available; MRI—Magnetic Resonance Imaging; N/d—Not defined; HMD—Head-Mounted Display; OTS—Optical Tracking System; CT—Computed Tomography; TRE—Target Registration Error.

## Data Availability

Data are contained within the article.

## Appendix A

Search	(Augmented reality OR AR) AND (skull-base)
Filter	none
Search details	(“augmented reality” [MeSH Terms] OR (“augmented” [All Fields] AND “reality” [All Fields]) OR “augmented reality” [All Fields]) AND (“skull base” [MeSH Terms] OR (“skull” [All Fields] AND “base” [All Fields]) OR “skull base” [All Fields])

## Appendix B

**Section/Topic**	**#**	**Checklist Item**	**Reported on Page #**
Title			
Title	1	Identify the report as a systematic review, meta-analysis, or both.	1
Abstract			
Structured summary	2	Provide a structured summary including, as applicable: background; objectives; data sources; study eligibility criteria, participants, and interventions; study appraisal and synthesis methods; results; limitations; conclusions and implications of key findings; systematic review registration number.	1
Introduction			
Rationale	3	Describe the rationale for the review in the context of what is already known.	2
Objectives	4	Provide an explicit statement of questions being addressed with reference to participants, interventions, comparisons, outcomes, and study design (PICOS).	2
Methods			
Protocol and registration	5	Indicate if a review protocol exists, if and where it can be accessed (e.g., Web address), and, if available, provide registration information including registration number.	2
Eligibility criteria	6	Specify study characteristics (e.g., PICOS, length of follow-up) and report characteristics (e.g., years considered, language, publication status) used as criteria for eligibility, giving rationale.	3
Information sources	7	Describe all information sources (e.g., databases with dates of coverage, contact with study authors to identify additional studies) in the search and date last searched.	3
Search	8	Present full electronic search strategy for at least one database, including any limits used, such that it could be repeated.	Appendix A
Study selection	9	State the process for selecting studies (i.e., screening, eligibility, included in systematic review, and, if applicable, included in the meta-analysis).	3
Data collection process	10	Describe method of data extraction from reports (e.g., piloted forms, independently, in duplicate) and any processes for obtaining and confirming data from investigators.	3
Data items	11	List and define all variables for which data were sought (e.g., PICOS, funding sources) and any assumptions and simplifications made.	3
Risk of bias in individual studies	12	Describe methods used for assessing risk of bias of individual studies (including specification of whether this was done at the study or outcome level), and how this information is to be used in any data synthesis.	N/A
Summary measures	13	State the principal summary measures (e.g., risk ratio, difference in means).	N/A
Synthesis of results	14	Describe the methods of handling data and combining results of studies, if done, including measures of consistency (e.g., I^2^) for each meta-analysis.	N/A
Risk of bias across studies	15	Specify any assessment of risk of bias that may affect the cumulative evidence (e.g., publication bias, selective reporting within studies).	N/A
Additional analyses	16	Describe methods of additional analyses (e.g., sensitivity or subgroup analyses, meta-regression), if done, indicating which were pre-specified.	N/A
Results			
Study selection	17	Give numbers of studies screened, assessed for eligibility, and included in the review, with reasons for exclusions at each stage, ideally with a flow diagram.	Figure 1
Study characteristics	18	For each study, present characteristics for which data were extracted (e.g., study size, PICOS, follow-up period) and provide the citations.	3
Risk of bias within studies	19	Present data on risk of bias of each study and, if available, any outcome level assessment (see item 12).	N/A
Results of individual studies	20	For all outcomes considered (benefits or harms), present, for each study: (a) simple summary data for each intervention group (b) effect estimates and confidence intervals, ideally with a forest plot.	Table 1, Table 2 and Table 3
Synthesis of results	21	Present results of each meta-analysis done, including confidence intervals and measures of consistency.	N/A
Risk of bias across studies	22	Present results of any assessment of risk of bias across studies (see Item 15).	N/A
Additional analysis	23	Give results of additional analyses, if done (e.g., sensitivity or subgroup analyses, meta-regression [see Item 16]).	N/A
Discussion			
Summary of evidence	24	Summarize the main findings including the strength of evidence for each main outcome; consider their relevance to key groups (e.g., healthcare providers, users, and policy makers).	4–8
Limitations	25	Discuss limitations at study and outcome level (e.g., risk of bias), and at review level (e.g., incomplete retrieval of identified research, reporting bias).	10
Conclusions	26	Provide a general interpretation of the results in the context of other evidence, and implications for future research.	11
Funding			
Funding	27	Describe sources of funding for the systematic review and other support (e.g., supply of data); role of funders for the systematic review.	11
